# Sirolimus-based induction therapy for lupus nephritis: A single-arm, open-label pilot clinical trial

**DOI:** 10.1515/jtim-2026-0019

**Published:** 2026-02-18

**Authors:** Bo Zou, Pingting Yang

**Affiliations:** Department of Rheumatology and Immunology, The First Hospital of China Medical University, Shenyang, Liaoning Province, China

## To the editor

Systemic lupus erythematosus (SLE) is a chronic autoimmune disease that predominantly affects women of childbearing age and poses significant long-term health challenges. Among its diverse manifestations, lupus nephritis (LN) stands out because it occurs in approximately 40–50% of patients and is closely linked to progressive renal impairment and high morbidity.^[1]^ Conventional management of LN generally relies on high-dose corticosteroids in combination with immunosuppressive agents such as cyclophosphamide (CYC), mycophenolate mofetil, and tacrolimus. However, the efficacy of these regimens is often limited by suboptimal remission rates and significant adverse effects—including gonadal toxicity, bone marrow suppression, and nephrotoxicity—which compromise their long-term use.^[2]^ While B-cell-targeted therapies represent a promising therapeutic advance, their prohibitive cost restricts widespread access, particularly in resource-limited settings. Therefore, given the marked clinical heterogeneity of SLE, there is a pressing need to develop optimized and individualized therapeutic strategies that balance efficacy, safety, and cost-effectiveness.

The mammalian target of rapamycin (mTOR) pathway has emerged as a key player in the pathogenesis of SLE, with its activation contributing to aberrant immune cell function.^[3,4^] Sirolimus (rapamycin), an mTOR inhibitor, has demonstrated efficacy in preclinical lupus models by suppressing autoimmunity and preventing nephritis.^[5,6]^ Clinical studies and real-world data have suggested that sirolimus is effective and well-tolerated in SLE patients, including those with LN, potentially reducing disease activity and steroid requirements.^[7,8]^ Building on these findings, this study evaluates a novel multi-target regimen for LN, combining sirolimus with low-dose CYC and corticosteroids. This strategy aims to target multiple pathogenic mechanisms simultaneously, potentially enhancing therapeutic efficacy while minimizing adverse drug reactions associated with high-dose immunosuppression.

This single-arm, open-label trial enrolled fifteen adult patients (aged 18–65 years) with confirmed SLE diagnosed according to the 2012 Systemic Lupus International Collaborating Clinics criteria. Inclusion criteria included proteinuria ≥1.5 g/d and a Systemic Lupus Erythematosus Disease Activity Index (SLEDAI) score ≥8. Patients with renal dysfunction attributable to hypertensive or diabetic nephropathy were excluded. The study was approved by the Ethics Committee of the First Affiliated Hospital of China Medical University (2019-236-2) and registered with the Chinese Clinical Trial Registry (ChiCTR1900027957).

The treatment regimen consisted of intravenous methylprednisolone sodium succinate (0.25 g/d) for 3 days, followed by oral prednisone acetate (initial dose 0.8 mg/kg/d) with gradual tapering to a maintenance dose. Sirolimus (1 mg daily) was initiated concurrently, with adjustments based on blood levels (target 5–10 μg/L). Intravenous CYC was administered at 0.4 g every two weeks for four sessions over two months.

Patients underwent systematic assessments at weeks 4, 12, and 24. The primary endpoint was the proportion of patients achieving a complete response (CR) or partial response (PR) at 24 weeks. CR was defined as proteinuria < 0.5 g/d, serum albumin ≥3.5 g/dL, and stable renal function. PR was defined as proteinuria < 3.5 g/d (reduction > 50% from baseline), serum albumin ≥3.0 g/dL, and stable renal function. Non-responders (NR) did not meet CR or PR criteria.^[9]^ Secondary outcomes included renal function (serum creatinine, estimated glomerular filtration rate), proteinuria, urinary red blood cell count, serum albumin, complement components C3 and C4, SLEDAI, and autoantibody positivity rates. Safety assessments included detailed patient histories, physical examinations, laboratory evaluations, and monitoring of sirolimus blood concentrations and dosages, with systematic documentation and grading of treatment-related adverse events (TRAEs).

Quantitative variables were analyzed using medians and interquartile ranges (IQR). Qualitative variables were expressed as frequencies and proportions. Fisher’s exact test or Cochran’s Q test was used for comparing proportions, and the Friedman test for multiple sample comparisons over time. Statistical analyses were performed using IBM SPSS Statistics, version 26, with a *P*-value < 0.05 considered statistically significant.

Between November 2019 and October 2023, 15 eligible patients were enrolled (Supplementary Table S1). At week 24, 13 patients had completed the trial; two patients withdrew due to TRAEs and were considered non-responders in the intention-to-treat analysis. Accordingly, the overall response rate in the intention-to-treat population was 73.3% (11/15), comprising 5 CR and 6 PR (Supplementary Figure S1A). The median daily prednisone dose at week 24 was 7.5 mg (IQR 7.5 to 10 mg) (Supplementary Figure S1B). Compared to baseline, significant improvements were observed at week 24 in complement levels, anti-dsDNA antibody positivity, proteinuria, serum albumin, estimated glomerular filtration rate (eGFR), and SLEDAI score (all *P* < 0.001) (Supplementary Figure S1C).

All patients who achieved CR were anti-dsDNA positive, including five at the week-24 endpoint and two additional patients at weeks 32 and 48 during extended follow-up. Patient 10 was the only anti-dsDNA–positive individual who did not achieve CR. Patient 10 (NR with modified treatment at week 48), patient 8 (NR), and patient 5 (discontinued) presented with thrombocytopenia, thrombotic microangiopathies, and antiphospholipid syndrome (APS), respectively. These unsatisfactory outcomes, despite appropriate anticoagulation, suggest that APS or potential APS may be resistant to sirolimus.

Patient 5 (APS) discontinued treatment due to worsening renal function. Patient 11 developed *Pneumocystis jirovecii* pneumonia (PJP), requiring hospitalization and subsequent withdrawal from the study, despite successful trimethoprim-sulfamethoxazole treatment. Both patients who withdrew had a long disease duration, confirmed or suspected APS, and poor baseline renal function.

A total of 14 TRAEs were observed, with infections being the most common (35.7%). All infections occurred exclusively during the initial 12 weeks of intensive immunosuppressive treatment. Infections comprised one PJP, two herpes zoster episodes, one upper respiratory infection, and one herpes simplex (Supplementary Figure S1D). After completing the study, patients 1 and 6 reported pregnancies at 54 and 117 weeks, respectively.

In this single-center, open-label pilot trial, we evaluated a novel induction regimen combining sirolimus with low-dose CYC and corticosteroids in active LN. At 24 weeks, this multi-target strategy yielded significant clinical and laboratory improvements, including restoration of complement C3 and C4 levels, reduction in anti-dsDNA antibody titers, decreased proteinuria, increased serum albumin, and improved eGFR. To place our efficacy findings in the context of existing evidence, the overall response rate in our study (73.3%) was comparable to the 75.0% reported for the intravenous CYC arm in a large, multicenter randomized clinical trial involving a similar patient population.^[9]^ While acknowledging the inherent limitations of cross-study comparisons, this comparable efficacy, coupled with the potentially improved safety profile observed in our study, suggests that mTOR pathway modulation with sirolimus may be a promising addition to conventional induction therapy in LN.

A noteworthy finding was that CR was achieved predominantly in anti-dsDNA-positive patients, suggesting this subgroup is especially responsive to sirolimus-based therapy. In contrast, patients with APS or potential APS exhibited markedly poor responses, with a CR rate of only 33.3% at week 24—despite statistically significant improvements in disease activity indices (*e.g*., SLEDAI). These results indicate that APS patients may require alternative or adjunctive therapeutic strategies.

Regarding safety, the regimen was generally well tolerated, with most TRAEs classified as mild (grades 1–2). Infections were the most frequent complication, affecting 35.7% of patients during the initial 12 weeks of intensive immunosuppression. The occurrence of a serious opportunistic infection (PJP) in one patient underscores the critical need for rigorous monitoring and the potential benefit of prophylactic strategies during the early induction phase. Balancing immunosuppressive efficacy against infection risk remains a critical challenge in LN management.

This study has several limitations, including its single-center design, small sample size, and relatively short follow-up. Future research should therefore involve larger, multicenter randomized controlled trials with extended follow-up, complete biopsy information to allow for stratified analysis, refined sirolimus and CYC dosing protocols, and patient stratification based on autoantibody profiles and APS status to optimize individualized therapy.

This pilot study suggests that sirolimus-based induction therapy represents a feasible and effective approach for treating LN, particularly in patients positive for anti-dsDNA antibodies. Future randomized controlled trials are warranted to validate these findings, address the limitations of the current study, and investigate tailored strategies for high-risk subgroups (*e.g*., patients with APS) to optimize the efficacy-safety balance for managing LN.

## Supplementary Material

Supplementary Material Details

## References

[1] Mok CC, Teng YKO, Saxena R, Tanaka Y (2023). Treatment of lupus nephritis: consensus, evidence and perspectives. Nat Rev Rheumatol.

[2] Kidney Disease (2024). Improving Global Outcomes (KDIGO) Lupus Nephritis Work Group. KDIGO 2024 clinical practice guideline for the management of lupus nephritis. Kidney Int.

[3] Oaks Z, Winans T, Huang N, Banki K, Perl A (2016). Activation of the mechanistic target of rapamycin in SLE: explosion of evidence in the last five years. Curr Rheumatol Rep.

[4] Murayama G, Chiba A, Kuga T, Makiyama A, Yamaji K, Tamura N (2020). Inhibition of mTOR suppresses IFNα production and the STING pathway in monocytes from systemic lupus erythematosus patients. Rheumatology (Oxford).

[5] Warner LM, Adams LM, Sehgal SN (1994). Rapamycin prolongs survival and arrests pathophysiologic changes in murine systemic lupus erythematosus. Arthritis Rheum.

[6] Lui SL, Yung S, Tsang R, Chan KW, Tam S, Chan TM (2008). Rapamycin prevents the development of nephritis in lupus-prone NZB/W F1 mice. Lupus.

[7] Eriksson P, Wallin P, Sjöwall C (2019). Clinical experience of sirolimus regarding efficacy and safety in systemic lupus erythematosus. Front Pharmacol.

[8] Lai ZW, Kelly R, Winans T, Marchena I, Shadakshari A, Yu J (2018). Sirolimus in patients with clinically active systemic lupus erythematosus resistant to or intolerant of conventional medications: a single-arm, open-label, phase 1/2 trial. Lancet.

[9] Zheng Z, Zhang H, Peng X, Zhang C, Xing C, Xu G (2022). Effect of tacrolimus vs intravenous cyclophosphamide on complete or partial response in patients with lupus nephritis: a randomized clinical trial. JAMA Netw Open.

