# Supplementary material for: Sirolimus-based induction therapy for lupus nephritis: A single-arm, open-label pilot clinical trial
Source: J Transl Int Med. 2026 Feb 18;14(2):319–21. doi: 10.1515/jtim-2026-0019 (PMC13110455; doi:10.1515/jtim-2026-0019)
Supplement: Supplementary file 1 — Supplementary Material Details [file jtim-2026-0019_sm.pdf]

Supplementary materials

A

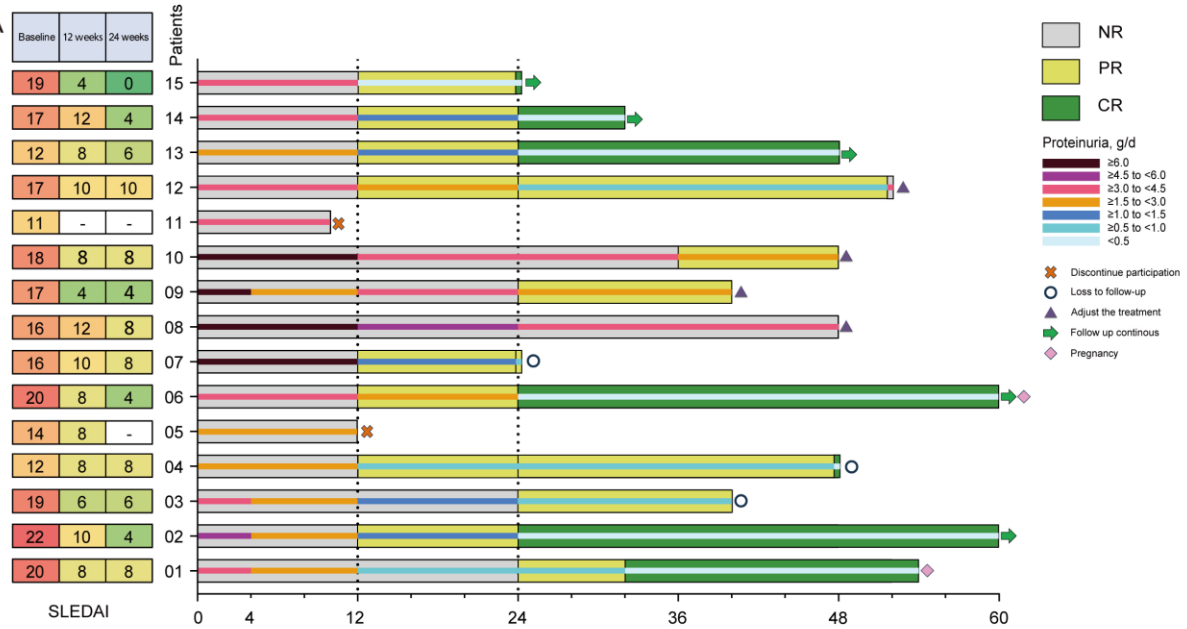

B

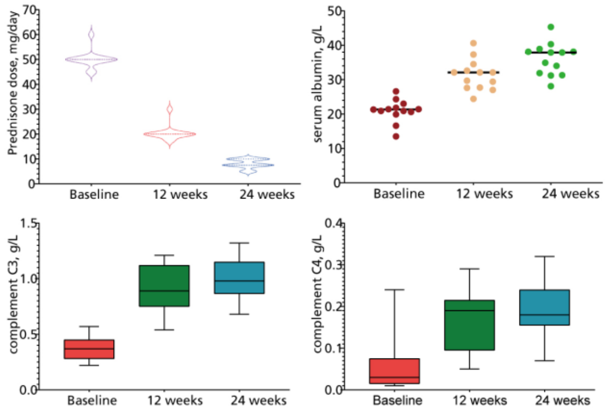

C

Parameters	Baseline	12 weeks	24 weeks	*P*
SLEDAI	17(16-19.5)	8(7-10)	6(4-8)	0.000
Creatinine, umol/L	90(70-116)	63(52-76)	62(55-70)	0.001
eGFR, mL/min/1.73 m^2	76.6(53.2-102.3)	109.8(84.7-124.8)	114.2(92.8-124.4)	0.001
Alb, g/L	21.4(20.3-23.7)	32.1(27.7-35.9)	37.9(31.6-39.6)	0.000
Proteinuria, g/d	3.999(3.190-6.369)	1.238(0.897-2.343)	0.599(0.347-1.662)	0.000
RBCs/HPF	82.2(43.2-145.6)	11.8(7.7-23.4)	13.8(6.9-22.1)	0.000
C3, g/L	0.37(0.28-0.45)	0.89(0.75-1.12)	0.98(0.87-1.15)	0.000
C4, g/L	0.03(0.02-0.08)	0.19(0.10-0.22)	0.18(0.16-0.24)	0.000
Low C3, %(*n*)	92.3%(12/13)	23.1%(3/13)	7.7%(1/13)	
Low C4, %(*n*)	84.6%(11/13)	23.1%(3/13)	7.7%(1/13)	
DsDNA, %(*n*)	61.5%(8/13)	15.4%(2/13)	9.1%(1/11)	0.012
Smith, %(*n*)	23.1%(3/13)	15.4%(2/13)	9.1%(1/11)	0.856
U1RNP, %(*n*)	30.7%(4/13)	23.1%(3/13)	18.2%(2/11)	0.892
AnuA, %(*n*)	61.5%(8/13)	30.8%(4/13)	0%(0/11)	0.006
AHA, %(*n*)	76.9%(10/13)	38.5%(5/13)	18.2%(2/11)	0.014
Rib-P, %(*n*)	38.5%(5/13)	30.8%(4/13)	27.3%(3/11)	0.909

Quantitative variables were summarized as medians and interquartile ranges. Statistical analyses were performed using the Friedman test or Fisher's Exact test to compare differences across baseline, 12 and 24 weeks.

D

Treatment-related adverse events	Cases	Grade
Weight gain	1	1
Gastrointestinal reactions	3	1
Menstrual disorder	2	1
Liver dysfunction	2	1-2
Upper respiratory tract infection	1	2
Herpes simplex	1	1
Varicella zoster virus	2	2
Pneumocystis jirovecii pneumonia	1	3
Increased creatinine	1	2

Supplementary Figure S1: Efficacy and safety of sirolimus-based therapy. (A) Treatment response, SLEDAI score, proteinuria, and follow-up data for all patients. (B, C) Clinical-laboratory parameters and statistical analysis. (D) Treatment-related adverse events as reported by the patients. NR, non-responders; PR, partial response; CR, complete response; eGFR, estimated glomerular filtration rate; Alb, serum albumin; Proteinuria, g/day; RBCs/HPF, urine red blood cells per high-power field; C3, complement C3; C4, complement C4; DsDNA, anti-double-stranded DNA antibodies; Smith, anti-Smith antibodies;

U1RNP, anti-U1 ribonucleoprotein antibodies; AnuA, anti-nucleosome antibodies; AHA, anti-histone antibodies; Rib-P, anti-ribosomal P protein antibodies.

Supplementary Table S1: Baseline Demographic and Clinical Characteristics of the Study Population, and Treatment Outcomes

Case	Gender/ Age (years)	Duration of SLE/LN (months)	Onset	DsDNA	C3 (g/L)	C4 (g/L)	Proteinuria (g/day)	RBCs /HPF	Alb (g/L)	Cr (umol /L)	eGFR	Other autoantibodies	Complications	Concomitant therapy	Outcome at week 24
**1**	F/ 24	0.5/0.5	I	2+	0.26	0.03	3.257	117	17	122	53.37	AnuA+	WBC↓, PLT↓	HCQ, Fosinopril	PR
**2**	F/ 28	0.5/0.5	I	+	0.37	0.02	4.518	55.5	29	62	117.63	AnuA+, AHA+, Rib-P3+, MPO-ANCA+, β2GP1 +	APS, WBC↓, Class IV+V (AI 8, CI 1)	HCQ, Valsartan, Aspirin	CR
**3**	F/ 33	3/3	I	-	0.4	0.01	3.122	22.7	21	73	93.22	AnuA2+, AHA2+	WBC↓	HCQ	PR
**4**	F/ 42	1/1	I	+	0.57	0.06	2.797	82.2	24	54	111.76	Rib-P 3+	Class IV+V (AI 7, CI 0)	Fosinopril	PR
**5**	F/ 28	168/108	R	-	0.41	0.08	2.995	39.2	26	172	34.26	U1RNP3+, Sm3+, AnuA+, AHA3+, LAC and β2GP1+,	APS, AIHA	HCQ, Warfarin	Disc
**6**	F/ 35	36/36	R	+	0.45	0.09	4.07	166	21	79	83.55	AHA2+, Rib-P 2+	Class IV (AI 9, CI 3)	HCQ, Olmesartan	CR
**7**	F/ 25	1/1	I	-	0.3	0.01	6.735	178	21	66	111.39	Sm+, AnuA2+, AHA2+	Class IV-G(A) (AI 18, CI 0)	HCQ, Valsartan	PR
**8**	M/ 30	24/1.5	R	-	0.36	0.11	8.33	117	14	121	68.79	U1RNP3+, Sm3+, AHA2+	TMA? , Class IV (AI 10, CI 2)	HCQ, Low-molecular-weight heparin , Telmisartan	NR
**9**	F/ 55	24/24	R	-	0.55	0.24	6.002	10.2	21	150	33.44	U1RNP+, AnuA+, AHA+	WBC↓	HCQ, Valsartan	PR
**10**	F/ 31	1/1	I	2+	0.24	0.03	9.053	82.4	20	110	57.59	AnuA2+, AHA1+	WBC↓, PLT↓	HCQ, Fosinopril	NR
**11**	F/ 58	216/60	R	-	0.74	0.07	4.202	1.95	32	148	33.28	U1RNP3+, β2GP1 +		HCQ, Valsartan, Aspirin	Disc
**12**	F/ 49	2/2	I	-	0.37	0.01	3.999	125	23	108	51.89	U1RNP3+, Sm3+, AnuA2+, AHA+, p-ANCA+,	WBC↓	HCQ, Valsartan	PR
**13**	F/ 51	36/19	R	2+	0.45	0.02	1.686	35.5	21	105	52.94	AHA+	AIHA	HCQ, Valsartan	CR
**14**	F/ 34	0.5/0.5	I	2+	0.22	0.04	3.499	50.9	22	74	91.06	Rib-P 3+, p-ANCA+,	WBC↓, Class IV -G(A)+V (AI 12, CI 0)	HCQ, Valsartan	CR
**15**	F/ 25	0.5/0.5	I	2+	0.35	0.04	3.372	199	27	90	76.56	U1RNP+, Sm+, Anu2+, AHA2+, Rib-P 3+	WBC↓, PLT↓, EBV	HCQ	CR

DsDNA, anti-double-stranded DNA antibodies; C3, complement C3; C4, complement C4; RBCs/HPF, urine red blood cells per high-power field; Alb, serum albumin; Cr, serum creatinine; eGFR, estimated glomerular filtration rate (mL/min/1.73m^2); I, initial; R, recurrence; APS, antiphospholipid syndrome; AIHA, autoimmune hemolytic anemia; TMA, thrombotic microangiopathy; WBC↓, leukopenia; PLT↓, thrombocytopenia; HCQ, hydroxychloroquine; AnuA, anti-nucleosome antibodies; AHA, anti-histone antibodies; U1RNP, anti-U1 ribonucleoprotein antibodies; Sm, anti-Sm antibodies; Rib-P, anti-ribosomal P

protein antibodies; β2GP1, anti-beta-2 glycoprotein 1 antibodies; LAC, lupus anticoagulant; ANCA, antineutrophil cytoplasmic autoantibodies; EBV, Epstein-Barr virus infection; AI, activity index; CI, chronicity index; CR, complete response; PR, partial response; NR, non-responders; Disc, discontinuation.
